# Preliminary studies on local anesthetic and antipyretic activities of *Spilanthes acmella* Murr. in experimental animal models

**DOI:** 10.4103/0253-7613.70106

**Published:** 2010-10

**Authors:** A. Chakraborty, B.R.K. Devi, R. Sanjebam, S. Khumbong, I.S. Thokchom

**Affiliations:** Vydehi Institute of Medical Sciences, Bangalore - 560 066, India; 1 Regional Institute of Medical Sciences, Imphal - 795 004, India

**Keywords:** Anti-pyretic, aspirin, local anesthetic, *Spilanthes acmella*, xylocaine

## Abstract

**Objective:**

*Spilanthes acmella* Murr. (Family: Compositae) is a herb that grows throughout the tropics. It is used in the treatment of rheumatism, fever, sore throat, and hemorrhoids. A tincture of the flowers is used to relieve toothache. The leaves and flowers produce numbness of the tongue when eaten as salad. The present study was undertaken to evaluate the local anesthetic and antipyretic activities of *S. acmella* in experimental animal models.

**Materials and Methods:**

Aqueous extract of *S. acmella* Murr. (SAM) was tested for local anesthetic action by (i) intracutaneous wheal in guinea pigs and (ii) plexus anesthesia in frogs. In both the models, 2% xylocaine was used as the standard drug. The anti-pyretic activity was determined by yeast-induced pyrexia in rats. Aspirin 300 mg/kg was used as the standard drug.

**Result:**

The test drug in concentrations of 10% and 20% produced 70.36% and 87.02% anesthesia respectively by the intracutaneous wheal compared to 97.22% anesthetic effect produced by 2% xylocaine (*P*<0.001). The mean onset of anesthesia with the test drug was 5.33±0.57 min compared to 2.75±0.31 min (*P*<0.001) for the standard drug in the plexus anesthesia model. In the anti-pyretic model, ASA in doses of 100, 200, and 400 mg produced dose-dependent reduction in mean temperature at various hours of observation.

**Conclusion:**

The present study shows that SAM has significant local anesthetic and antipyretic activities.

## Introduction

*Spilanthes acmella* Murr. (para -cress or toothache plant) is an indigenous herb of the family Compositae. It is called Pirazha in Assamese, Akarkara in Bengali, Maanja-lei in Manipuri, and Maratitige in Telegu. It grows as an annual herb throughout the tropics.[1,2] It has conical small yellow flowers. The whole plant is claimed to possess medicinal properties.[3,4] It is used in the treatment of rheumatism, fever, sore throat, and hemorrhoids. The flowers are chewed to relieve toothache. They produce redness of gums and increase salivation.[5,6] The leaves are eaten raw or as a vegetable in different parts of the world. When eaten, they produce tingling and numbness of the tongue. The crushed leaves are used to stupefy fish.[7,8] This study was undertaken to evaluate the local anesthetic activity of the aqueous extract of *S. acmella* Murr. (SAM) by (i) intracutaneous wheal in guinea pig; (ii) plexus anesthesia in frogs, and (iii) antipyretic activity by yeast-induced pyrexia in albino rats.

## Materials and Methods

### Preparation of the extract

Fresh aerial parts of *S. acmella* were collected, identified, and authenticated. They were cleaned, dried under shade, and powdered by a mechanical grinder. Sixty grams of the pulverized plant parts were extracted with distilled water using a soxhlet apparatus. The yield was 13.5% in the powder form.

### Phytochemical Studies

Freshly prepared SAM extract was subjected to phytochemical screening tests.[9] The chemical investigation with Wagner’s reagent and 5% dilute ferric chloride solution showed the presence of alkaloids, phenolic compounds, and tannins in the extract.

### Animals

Fully grown male guinea pigs (300 to 400 g), frogs, and albino rats (150-200 g) were procured from the central animal house of the institute. The animals were housed at controlled room temperature (24±2°C; relative humidity 60-70%) in a 12 h light-dark cycle. They were given standard laboratory diet and water ad libitum. The experimental protocol was approved by the Institutional Animal Ethics Committee. (IAEC)

### Drugs

The following chemicals were used: xylocaine (AstraZeneca), sodium chloride (Triveni chemicals), dried yeast, and acetyl salicylic acid.

### Acute Toxicity Study

No adverse effect or mortality was detected in albino rats up to 3 g/kg, p.o. of SAM during the 24 h observation period.[10]

### Local Anesthetic Activity

### 1. Intracutaneous wheal in guinea pigs

The animals were divided into four groups [Table 1]. On the day prior to the study, the hair on the back of guinea pigs near the midline (four different areas of 4 cm each) were clipped and removed. The drugs were injected intracutaneously in equal volumes of 0.2 ml into the shaved areas and wheals were marked with ink and the time of injection noted. The normal responses of the animals were observed first by applying pin pricks in the midline. Six pin pricks were then given uniformly every five min at an interval of four seconds on the wheal areas. The responses were recorded up to 30 min. A localized skin twitch, usually accompanied by squeak, was considered as the normal response to pin prick. When the animal failed to respond either by twitching of the muscle or squeaking following a pin prick, a negative response was recorded.[11,12]

**Table 1:** Local anesthetic activity of *Spilanthes acmella* Murr on intracutaneous wheal in guinea pigs

*Group*	*Drug*	*No. of negative response*	*Mean % failure of response*
A (Control)	0.9% saline	1.5 ± 0.76	4.16
B (Test)	10% SAM	25.33 ± 3.27*	70.36*
C (Test)	20% SAM	31.33 ± 1.08*	87.02*
D (Standard)	2% xylocaine	35.00 ± 0.51*	97.22*

* *P*<0.001 when compared to control; n=6 in each group

### 2. Plexus anesthesia in frogs

The frogs were divided into three groups [Table 2]. They were decerebrated and upper parts of their spinal cords were destroyed using a pithing needle. The abdominal viscera were excised and removed through a transverse incision made just below the sternum thereby forming a pouch. The lumbar plexus was exposed carefully without damaging it. The frogs were pinned to vertical boards with their legs hanging down. The drugs were administered into the abdominal pouch in sufficient volumes to submerge the lumbar plexus. The left and right limbs of the frogs were immersed every minute for maximum period of 10 s in beakers containing 0.1(N) HCL and normal saline, respectively. Afterwards the feet were rinsed in water. The time taken by the animals failing to withdraw their feet was recorded as the “onset of local anesthetic action.”[13–17]

**Table 2:** Local anesthetic activity of *Spilanthes acmella* Murr on plexus anesthesia in frogs

*Group*	*Drug*	*Onset of local anesthetic action (mean ±SEM) (min)*
A (control)	0.9% saline	24.16 ± 1.54
B (test)	20% SAM)	5.33 ± 0.57*
C (standard)	2% xylocaine	2.75 ± 0.31*

* *P*<0.001 when compared to control; n=6 in each group

### 3. Anti-pyretic activity

**Figure 1:**
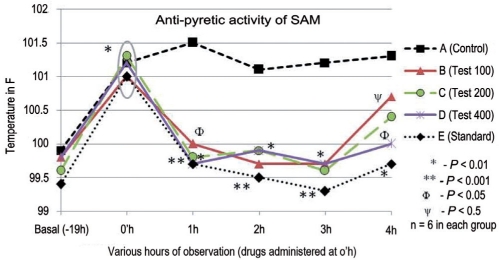
Antipyretic activity of *Spilanthes acmella* Murr in albino rats Anti-pyretic activity of SAM A: Control, B: Test 100mg, C: Test 200mg; D: Test 400 mg; E: Standard (Aspirin); * *P* < 0.01, when compared to initial basal temperature; ** *P*< 0.001, when compared to control at that hour; Ф *P* < 0.05, when compared to control at that hour; ψ *P* < 0.5, when compared to control at that hour; n = 6 in each group

Initial basal rectal temperatures of the animals were measured. The animals were then given sub-cutaneous injection of 20% aqueous suspension of dried yeast in 2% gum acacia at a dose of 20 ml/kg below the nape of the neck. After 19 h of yeast injection, the animals were restrained in individual cages for recording their rectal temperatures. Rectal temperatures were obtained by insertion of digital clinical thermometer. Thermometer was inserted to a constant depth of 3 cm. The animals were then grouped into five groups with six animals in each group [Figure 1]. The drugs were suspended in 2% gum acacia and administered orally. Temperature was recorded at hourly intervals up to 23 h after yeast injection.[18]

### Statistical Analysis

The results were analyzed for statistical significance by one-way ANOVA followed by Dunnet’s‘t’ test. A ‘*P*’ value of < 0.05 was considered significant.

## Results

In intracutaneous wheal model in guinea pigs, the test drug in a concentration of 10% and 20% produced 70.36% and 87.02% anesthesia compared to 97.22% anesthetic effect produced by 2% xylocaine. The negative responses of the standard and test groups showed a highly significant increase when compared to the control group. An increased concentration of the test drug produced an increase in local anesthetic activity (r=1) [Table 1].

The mean onset of anesthesia with the test drug was 5.33±0.57 min compared to 2.75±0.31 min for the standard drug. The onset of local anesthetic activity in the test and standard groups was significantly different from the control group [Table 2]. The anesthetic action of the standard and test drugs continued till 30 min of our observation period.

In the antipyretic model, SAM in doses of 100, 200, and 400 mg/kg reduced the temperature of pyretic rats significantly from first h to the third h, respectively (*P*<0.05-0.01). However, the reduction in pyrexia at the fourthh was not found to be significant. Aspirin lowered the temperature significantly throughout the observation period [Figure 1].

## Discussion

The local anesthetic activity of SAM was studied by the methods described by Burn *et al*., with slight modifications on(i) intracutaneous wheal in guinea pigs and (ii) plexus anesthesia in frogs. In the present study, 2% xylocaine was used as the standard drug in both the models, whereas the above workers used nupercaine as the standard in the intracutaneous wheal model and cocaine in the plexus anesthesia model. The wheal model is suitable for estimating the degree of anesthesia and its duration simultaneously, whereas the plexus anesthesia determines the onset of anesthesia.[17,19] The mean onset of local anesthetic action with SAM in concentration of 20% was 5.33±0.57 min (*P*<0.001). The anesthetic action continued till 30 min of the observation period. The findings suggest that SAM possesses a significant local anesthetic property. The antipyretic activity was studied by an yeast-induced method of Brownlee. For the production of pyrexia, yeast is widely used. Various workers used different concentrations and different doses of yeast. The mean initial basal rectal temperature in this study was 99.4±0.42 to 99.9±0.24° F. The rise in temperature after 19 h of induction was 101.0±0.37 to 101.3±0.32° F. The induction results correspond well with the available literature.[20,21] In this study, SAM reduced temperature of pyretic rats significantly from first h to the third h, respectively. Preliminary phytochemical studies showed the presence of flavonoids. There are reports that some flavonoids are predominant inhibitors of either cyclo-oxygenase or lipo-oxygenase.[22,23] Flavonoids are hydrolyzed by zaliva to deliver aglycones that have protective effect in the oral cavity.[24] Some workers have reported that *S. acmella* contains alkylamides.[25,26] The local anesthetic property of SAM observed in the study could be due to the presence of alkylamides. The antipyretic activity *S. acmella* demonstrated in the present study could be due to the presence of flavonoids.
